# Effects of Rumen-Protected Methionine Supplementation on Growth Performance, Nutrient Digestion, Nitrogen Utilisation and Plasma Amino Acid Profiles of Liaoning Cashmere Goats

**DOI:** 10.3390/ani13192995

**Published:** 2023-09-22

**Authors:** Wennan Wang, Lisha Ye, Xingtang Dou, Haiying Liu, Di Han

**Affiliations:** 1 College of Animal Science and Veterinary Medicine, Shenyang Agricultural University, Shenyang 110866, China; 18242141919@163.com (W.W.); 2022220568@stu.syau.edu.cn (L.Y.); 2 Liaoning Province Modern Agricultural Production Base and Construction Engineering Center, Liaoyang 111000, China; dou791120@sina.com; 3 Liaoning Cashmere Goat Breeding Farm Co., Ltd., Liaoyang 111000, China

**Keywords:** cashmere goats, rumen-protected methionine, amino acids, growth performance, nitrogen metabolism

## Abstract

**Simple Summary:**

Dietary methionine deficiency can limit the body growth and cashmere production of cashmere goats, leading to excessive nitrogen (N) excretion. This study demonstrated that rumen-protected methionine supplementation improved the growth performance of cashmere goats and reduced their manure N excretions and plasma urea N levels during the seasonal growth of cashmere fibres. The formulation of diets supplemented with rumen-protected methionine can enhance the utilisation of N and maximise the production capacity of cashmere goats.

**Abstract:**

This study determined the effects of rumen-protected methionine (RPM) supplementation on the growth performance, nutrient digestibility, nitrogen (N) utilisation and plasma amino acid profiles of Liaoning cashmere goats during cashmere fibre growth. Twenty-four yearling male cashmere goats (body weight: 35.41 ± 1.13 kg) were randomly assigned to four dietary treatments: a corn–soybean meal basal diet deficient in methionine (negative control, NC) and a basal diet supplemented with 1, 2 and 3 g/kg of RPM. The RPM supplementation quadratically increased the average daily gain (ADG) and decreased the feed to gain ratio (*p* = 0.001) without affecting the final body weight and dry matter intake. In particular, compared to NC, 2 g/kg RPM supplementation increased the ADG by 35 g/d (*p* < 0.001) and resulted in the lowest feed to gain ratio (*p* < 0.001). RPM increased the apparent total tract digestibility of N and decreased the faecal N levels, both in a linear fashion (*p* = 0.005). Urinary N levels did not have an effect, but the N retention levels increased linearly with PRM (*p* = 0.032). Moreover, the RPM decreased the plasma urea N levels (*p* < 0.001) and increased the plasma Met levels quadratically (*p* < 0.001). In conclusion, RPM supplementation in the diet of cashmere goats can enhance the utilisation of N and improve ADG during the cashmere fibre growing period, and 2 g/kg of RPM in the diet is suggested.

## 1. Introduction

Methionine (Met) is one of the essential amino acids (EAAs) in animals and plays an important metabolic and functional role. It is also the most limiting amino acid (AA) that stimulates the growth of cashmere, wool and mohair fibre [1,2,3]. When the supply of Met to animals is insufficient, it will limit the synthesis of body proteins and cashmere fibre keratin, leading to the supply of an AA surplus relative to the requirement [3,4]. Furthermore, surplus AAs are catabolized in the liver, and their nitrogen (N) content is converted into urea, with a large proportion being excreted via urine [5]. The data from monogastric animals showed that insufficient Met in the diet can reduce the digestibility of protein in the small intestine and increase the faecal N output [6]. The N in faeces and urine is gradually converted into nitrate, ammonia and nitrous oxide during the storage process, which contribute to air, soil and water pollution, have adverse effects on the environment and lead to global warming [7,8]. In addition to having adverse effects on the environment, the loss of dietary N in manure is also a significant economic loss for livestock farms, as protein is the most expensive dietary nutrient [9]. Therefore, improving the N utilisation and environmental sustainability and enhancing the EAA supply, including Met, in low-protein diets to alleviate EAA deficiency is important in livestock production [10].

Liaoning Cashmere goat is a major goat breed being raised in North-Eastern China, known for its high net cashmere rate and cashmere yield [11]. The sulphur contents in cashmere fibre from goats is approximately 2.7–5.4%, and the higher the sulphur contents in cashmere fibre, the finer the cashmere fibre [12,13]. Therefore, sulphur-containing AAs are crucial nutrients for cashmere goats in harvesting high-quality fibres and achieving better growth performance [14]. In North-Eastern China, crop straw (i.e., grazing) and a corn–soybean mixture are the main components of cashmere goat diets, which usually lack Met, and the offered protein frequently falls short of the recommended nutritional requirements [15]. It has been observed that feeding rumen-protected Met (RPM) can improve the growth performance of goats [16], milk yield of dairy goats [17,18] and wool characteristics of sheep [19], as well as to improve N utilisation in goats [16]. However, in relation to cashmere goats, the impact of RPM on performance and nutrient utilisation is currently poorly understood.

Unlike wool and mohair, cashmere fibres are produced seasonally [20]. These changes vary along with nutritional conditions because of the seasonal nature of cashmere production [21]. During the fast growth period of cashmere fibres (i.e., August to November), cashmere goats may have a relatively higher demand for protein and sulphur-containing AAs. These nutritional requirements drive the growth performance of cashmere goats [13]. However, to date, there is limited information concerning the impact on bioavailability, e.g., plasma Met concentrations, N utilisation in cashmere goats, especially during the fast growth period of cashmere fibre. Therefore, this study aimed to determine the effects of RPM on the growth performance, nutrient digestion, manure N losses and plasma AA profiles of Liaoning cashmere goats during the fast growth period of cashmere fibre. The results we obtained will be potentially useful to the formulation of diets supplemented with RPM for maximising the production capacity of cashmere goats.

## 2. Materials and Methods

### 2.1. Animals, Experimental Design and Diet

All procedures were approved by the Institutional Animal Ethics and Welfare Committee of Shenyang Agricultural University, Shenyang, China (No. 201801018). Twenty-four yearling male Liaoning cashmere goats with an initial body weight (BW) of 35.41 ± 1.13 kg and age of 14 ± 0.62 month were selected and randomly assigned to 4 dietary treatments (*n* = 6), then subjected to a 16-week experiment. The goats were randomly assigned to four dietary treatments: a corn–soybean meal-based basal total mixed ration (TMR) deficient in Met (negative control, NC), and the NC diet supplemented with 1, 2 and 3 g/kg of RPM, named 1RPM, 2RPM and 3RPM, respectively. The RPM was obtained from Beijing Yahe Nutritive Co., Ltd. (Beijing, China) and contained 60% DL-Met. PRM was mixed to the concentrates. The diets with similar metabolisable energy and protein levels were complete mixtures of 25% concentrate and 75% forage. The ingredient and nutrient compositions are depicted in Table 1. The AA compositions (measured values) of the feeds given to the animals are shown in Table 2. The goats were housed in separate wooden pens (5.57 × 3.00 m) with slotted wooden floors in an open-sided barn. All animals had daily exercise and free access to drinking water. Goats were fed individually ad libitum with TMR. Diets were offered twice daily at 0800 h and 1600 h at ~110% of consumption on the preceding few days after refusals were collected and weighed.

### 2.2. Measurements

#### 2.2.1. Feed Analyses

Feed samples were collected every two days, and composite samples of the feedstuffs were prepared biweekly. The samples were dried in a forced-air oven at 55 °C for 48 h to determine the dry matter (DM); the samples were subsequently ground (Wiley Mill, Thomas Co.; Philadelphia, PA, USA) to pass through a 1 mm sieve and then stored at room temperature until analysis. The analyses included the determination of DM (method 934.01), N (Leco TruMac CN, St. Joseph, MI, USA; method 990.03), ash (method 930.15), calcium (method 96.08) and phosphorous (method 946.06) contents according to AOAC methods [22]. In addition, neutral detergent fibre (NDF) and acid detergent fibre (ADF) were analysed using an ANKOM200 Fibre Analyzer (ANKOM Technology Corp., Macedon, NY, USA) using the filter bag technique, which involved the addition of heat-stable α-amylase and sodium sulphite [23]. Following hydrolysis with 6 N HCl at 110 °C for 24 h, the AAs in the diet were assayed through ion-exchange chromatography using an automatic AA analyser (Hitachi L8800 Automatic Amino Acid Analyzer; Tokyo, Japan).

#### 2.2.2. Animal Performance

Body weight was recorded at the beginning and end of the experiment. The initial and final values were used to determine the ADG. The diet and that refused for individual goats were weighed every day throughout the experiment to determine average dry matter intake (DMI, g/d). Feed to gain ratio (F/G) was then calculated.

#### 2.2.3. Blood Sampling and Measurements

Blood samples were obtained on the tenth day of the experiment. The samples were drawn by jugular venipuncture in the morning before feeding and placed into 10 mL heparinized tubes. The plasma was harvested via centrifugation at 3000× *g* and 4 °C for 20 min. A portion of the plasma was stored at −20 °C until the analysis for urea N content [24]. A 2 mL sample was placed into a tube containing an equal volume of 5% yellow salicylic acid to precipitate the proteins; subsequently, the samples were centrifuged at 14,000× *g* and 4 °C for 30 min. Then, the supernatant was obtained to determine the plasma-free AA concentrations using an automatic AA analyser (Hitachi L8800 Automatic Amino Acid Analyzer, Tokyo, Japan).

#### 2.2.4. Faecal and Urine Analyses

Total faeces and urine collection and sampling were performed for five days in 0.6 m × 1.2 m metabolism cages during the fifth week. During this five-day period, feed refusals were also weighed and sampled daily. Faeces were collected in plastic mesh baskets placed under the floor of the metabolism cages to separate faeces and urine. Urine was collected using a funnel draining into plastic buckets containing 10 mL sulphuric acid (10%; vol/vol). Approximately 10% of faeces and urine and about 40 g of the orts were sampled daily to prepare composite samples for each goat. All samples were frozen at −20 °C until analysis. The faecal samples and orts of each goat were dried, ground and then analysed for DM, ash, NDF and ADF contents, as described above for the feed samples. The composited faecal and urine samples were also analysed for total N content using the Kjeldahl digestion method. Nutrient digestibility was based on feed intake, refusals and faeces over 5 d when faeces were collected. Retention N was the difference between N intake and faecal and urinary N.

### 2.3. Statistical Analysis

Data were analysed using the IBM SPSS 19.0 statistical software for windows (Statistical Package for Social Science; SPSS Inc., Chicago, IL, USA). The data were tested for normality and homogeneity of variances and showed a normal distribution. The effect of dietary RPM dose on the investigated parameters was analysed using one-way ANOVA followed by an honestly significant difference (HSD) test. The linear and quadratic contrasts for the relationships of the RPM doses with each parameter were determined. The treatment effects and the contrasts were considered statistically significant at *p* < 0.05. Data were presented as means ± standard error of the mean (SEM) and *p* values.

## 3. Results

In this study, different RPM doses were added to a corn–soybean meal-based diet to determine the impact of RPM supplementation on growth performance, blood AA profiles, the N excretions and N utilisation of cashmere goats during the fast growth period of cashmere fibres. The RPM contained 60% DL-Met. The NC group, 1RPM group, 2RPM group and 3RPM group were provided with 3.42, 4.13, 4.79 and 6.03 g Met/kg DM, respectively (Table 2).

### 3.1. Animal Performance

The effects of the dietary RPM dose on ADG, DMI and F/G mean values in cashmere goats are presented in Table 3. No differences were observed between the initial and final BW across the different RPM doses (*p* > 0.05). The ADG exhibited a linear and quadratic relationship with RPM dose (*p* = 0.013 and 0.001, respectively), wherein it increased from 70.64 g/d for the NC group to the highest 106.13 g/d for the 2RPM group. In addition, the RPM supplementation did not affect the DMI (*p* > 0.05). The NC group exhibited the highest F/G (13.75), which decreased linearly with RPM dose (*p* = 0.001).

There was a linear and quadratic relationship between RPM dose and both ADG and F/G (*p* < 0.01). Based on the quadratic regression equations, when the RPM dose was 1.81 g/kg, the ADG was highest (y = −9.18x^2^ + 33.29x + 67.82), whereas the F/G was the lowest when the RPM dose was 1.96 g/kg (y = 1.18x^2^ − 4.63x + 13.70). The corresponding Ra^2^ values were 0.760 and 0.996, respectively. Considering the HSD results and the quadratic model of ADG and F/G, we concluded that the 2RPM supplementation exhibited the best results for growth performance.

### 3.2. Nutrient Digestibility

The nutrient intake and apparent digestibility are presented in Table 4. In contrast to the average DMI during the entire experiment, the DMI during the faecal sample collection was not affected by the dietary treatments (*p* > 0.05). The total tract digestibility of DM and NDF and the intake of digested NDF were greater for the 2RPM group than for the other treatments (*p* = 0.036, 0.027 and 0.030, respectively). However, the intake of digested DM was not influenced by RPM dose (*p* > 0.05). The OM intake and the intake of digested OM were higher for the 2RPM group than for the other treatments (*p* < 0.05), and there was a quadratic relationship between both the OM intake and the intake of digested OM with RPM doses. The ADF intake, intake of digested ADF and total tract digestibility of ADF were similar across all treatments.

### 3.3. Nitrogen Retention

The intake of N, output N in faeces and urine, retention N and plasma urea N levels are presented in Table 5. Similar to DMI (Table 3), the N intake did not change with RPM dose (*p* > 0.05), whereas the intake of digested N and the total tract digestibility of N increased linearly with RPM dose (*p* = 0.015 and *p* < 0.01, respectively). Faecal N (g/d) level, expressed as a percentage of N intake, decreased linearly with RPM dose. Urinary N output (g/d or a percentage of N intake) was not affected by RPM dose (*p* > 0.05). Retention N (g/d or a percentage of N intake) increased linearly with RPM dose (*p* < 0.05); however, no difference among the treatments was observed when this parameter was expressed as a percentage of digestible N. Plasma urea N exhibited a positive linear and quadratic relationship with RPM dose (*p* < 0.001). Based on the quadratic regression equation y = 0.55x^2^ − 2.06x + 4.05 (Ra^2^ = 0.964), the plasma urea N level was lowest when the RPM dose was 1.87 g/kg.

### 3.4. Plasma-Free Amino Acids

The effects of RPM dose on plasma-free AA concentrations are presented in Table 6. The plasma Met concentration exhibited a linear and quadratic relationship with RPM dose (*p* < 0.001). The 1RPM, 2RPM and 3RPM treatments increased the plasma Met concentration by 3.88, 3.68 and 2.93 ng/mL relative to the NC, respectively (*p* < 0.001). Based on the regression calculation, y = −0.68x^2^ + 3.29x + 3.60 (Ra^2^ = 0.986), the plasma Met concentration was highest when the RPM dose was 2.42 g/kg. The concentration of plasma phenylalanine (Phe), lysine (Lys), leucine (Leu) and isoleucine (Ile) decreased linearly with the RPM dose (*p* < 0.05), while the total plasma EAA and non-essential AA (NEAA) concentrations also decreased linearly with the RPM dose. The branched-chain AA (BCAA) and total AA (TAA) concentrations were greater for the NC group than for the treatment groups (*p* < 0.01).

## 4. Discussion

Studies have shown that Met plays a specific role in protein metabolism and synthesis in the body and wool and is the primary limiting AA in furred animals, including cashmere goats [12,14]. Because Met deficiency may cause growth delays [13,25], this study investigated the effect of RPM supplementation in a Met-deficient diet on the growth performance and nutrient utilisation of cashmere goats. The findings indicated that ADG had a quadratic relationship with RPM level and increased 35.49 g/d for 2 g/kg RPM supplementation compared to the NC group during the 16-week feeding period. The ADG of 3RPM did not increase further relative to 2RPM, suggesting that 2RPM might have corrected the deficiency and helped goats meet the Met requirement. Moreover, the F/G was generally lower in the RPM groups than that in the NC group. The improvement in growth performance in goats may be due to increasing the Met availability in the small intestine to enhance the utilisation of N. Similar results were also reported, as feeding goats with 2.5 g RPM/kg DM improved their growth performance [16]. Another study reported a significant improvement in ADG and F/G when actively growing Tan lambs were fed a low-protein diet supplemented with 1.5 g/d of RPM at a 1.0 and 0.8 voluntary intake [26]. However, these findings are also inconsistent with some previous results. Obeidat et al. [27] did not achieve a significant improvement in performance and F/G in ram lambs supplemented with different RPM doses. Similarly, no significant differences in performance were observed in Shaimi goats and beef steers supplemented with RPM [28,29]. Based on the inconsistencies between the present results and previous findings, the positive effect of RPM supplementation on growth performance in our study suggested that the cashmere goats required additional Met to address Met deficiency in order to maximise their body growth and cashmere fibre production. Considering the HSD results and the quadratic model of ADG and F/G, the 2RPM supplementation seemed to exhibit better results for growth performance.

Consistent with previous findings, the DM intake was similar across the different RPM doses, both during the digestibility determination period and the entire experimental period, as all parameters were kept similar, except for the RPM dose [5,16]. The apparent total tract digestibility of DM, NDF and N was improved using the RPM supplementation. Baghbanzadeh-Nobari et al. [30] found that cows supplemented with DL-Met (1.2 g/kg DM) exhibited significantly increased apparent DM and ADF digestion. However, data on the relationship between nutrient digestibility and dietary RPM doses in ruminants remain limited. Early studies suggested that supplements with sulphur-containing AAs can better improve rumen microbial growth compared to supplements lacking these AAs [31]. Improved rumen microbial growth should lead to more efficient DM, OM and NDF disappearance. However, in supplementation with Met from a rumen-protected source, some Met remains available to the rumen microbes [32].

Nitrogen digestibility has often been assessed in ruminants receiving RPM in their diet. The data on dairy cows indicated that RPM supplementations at 0, 11.0, 19.3 and 27.5 g/d improved the apparent total tract digestibility of dietary N and decreased faecal N levels. Studies involving other species have indicated that Met supplementation can improve the AA balance in the diets, promote protein synthesis and enhance protein digestion and absorption by improving small intestine morphology and stimulating the production of digestive enzymes in the pancreas [4,16]. Similarly, urinary N excretion was not affected by RPM dose [5]. Owing to a significant decrease in the manure N excretion, the N retention linearly increased with RPM dose, indicating an improvement in protein utilisation efficiency in the diet. Reductions in faecal N outputs improve the environmental sustainability of livestock farming, as the transformation of manure N into nitrate, ammonia and nitrous oxide contributes to soil, air and water pollution [7,33].

Plasma urea N concentration is often used to assess the nutritional status of livestock in terms of AAs, which reflects the efficiency in protein synthesis and the urea recycling rate [34]. In our study, the plasma urea N concentrations significantly decreased, highlighting that whole-body N catabolism was affected by the RPM supplementation. The decrease in plasma urea N content indicates an increase in protein synthesis, whereas the opposite trend indicates an increase in protein catabolism and urea recirculating in the liver [35]. In the present study, the plasma urea N concentrations in the 1RPM, 2RPM and 3RPM groups were lower than in the NC group (2.73, 1.96 and 2.91 vs. 3.99 mmol/L, respectively), with an average concentration of 2.53 mmol/L. All groups fell within the normal range [34]. The regression analysis showed that the plasma urea N level was lowest when the RPM dose was 1.87 g/kg. Based on N utilisation resulting from the RPM dosage, the 2RPM treatment apparently satisfied the metabolic Met requirements of the investigated goats.

Plasma-free AA concentrations serve as an indicator of the amount of limiting AAs in the body, generally influenced by the composition of absorbed AAs [36]. In this study, Met concentrations increased linearly and quadratically with RPM dose. The plasma Met concentration in treatment groups fed a diet containing 4.13–6.03 g of Met/kg DM exhibited 6.45–7.40 ng/mL, whereas the plasma concentration in the NC group exhibited 3.52 ng/mL. The elevated plasma Met concentration indicated that the rumen-protected technology had successfully presented Met to the small intestine and that Met had been released for absorption [5]. Other studies have also reported an increased plasma Met concentration caused by supplementation with Met from rumen-protected sources [37,38,39,40,41]. The availability of free AAs in the small intestine can positively influence blood AA concentrations [32]; thus, the plasma Met concentration may be used to evaluate the duodenal flows of Met [42]. When adequate energy is available, protein deposition shows a linear relationship with the supply of the most limiting AA, until another AA becomes more limiting [43]. Inconsistent results have been reported regarding the ability of RPM supplementation to render Met available for absorption in the small intestine [44,45]. However, the present study showed that Met was absorbed, which affected the utilisation of other AAs. Based on the quadratic regression analysis, when the RPM dose was 2.4 g/kg DM, the plasma Met concentration was highest.

The plasma concentration of Lys, Phe, Leu and Ile decreased with the increase in RPM dose. Meanwhile, RPM supplementation had a negative impact on total plasma EAA, NEAA and TAA concentrations. These results suggested that the goats supplemented with RPM were more efficient in utilising Lys, Phe and two of the BCAAs (Leu, and Ile) than NC goats. These findings also indicated that cashmere goats supplemented with RPM had a more efficient utilisation of EAAs, NEAAs and TAAs, as the concentration of these AAs declined while the N retention increased on the 10th day. Toledo et al. [43] also observed that feeding RPM in multiparous Holstein cows during the periparturient period increased the plasma Met concentration by about 38%; however, the concentrations of Leu, Val, Asp, Ser and Tyr were significantly decreased, and the TAA concentration tended to decrease. However, King et al. [5] reported that supplementation of RPM (0, 11.0, 19.3 and 27.5 g/d) to a diet deficient in Met of dairy cows resulted in a linear increase in arterial concentration of Met, Lys, Try and Arg with RPM dose, while the BCAA concentration remained unchanged. The reason behind these inconsistent results is obscure. They could be attributable to differences in diet composition (i.e., some studies may have used treatment diets with insufficient Met, while others with sufficient Met), the dosage of supplementary RPM, and the age, breed and physiological status of the animals [46]. But more elaborate studies should be conducted to better understand their dynamic roles in the growth performance of cashmere goats.

## 5. Conclusions

The supplementation of RPM (0, 1, 2 and 3 g/kg) to a diet deficient in Met (4.13 g/kg DM) increased the ADG and decreased the F/G without affecting the final BW and DMI of cashmere goats. The average daily gain increased by 35 g/d for does of 2 g/kg RPM to the diet, but the rate of the increments declined for further supplementation of RPM. The RPM dose linearly decreased the faecal N excretion, indicating improved apparent total tract digestibility of N. Despite unaffected urinary N excretion, the retention N increased linearly while the plasma urea N level decreased quadratically with RPM dose. The plasma Met concentration increased quadratically with RPM does. Moreover, Lys, Leu and Ile concentrations decreased linearly with RPM dose, possibly due to the enhanced AA utilisation and N retention. Overall, the present RPM product providing 2 g/kg to the diet seemed to resolve the dietary Met deficiency of the cashmere goats, attenuated their faecal N excretions and improved the environmental sustainability.

## Figures and Tables

**Table 1:** Ingredient composition and nutrient levels of diets fed to yearling Liaoning cashmere goats (DM basis).

Items	Treatment ^1^			
	NC	1RPM	2RPM	3RPM
Ingredients, %				
Alfalfa	25.00	25.00	25.00	25.00
Corn stover	25.00	25.00	25.00	25.00
Peanut straw	25.00	25.00	25.00	25.00
Corn	12.10	12.20	12.30	12.40
Wheat bran	5.15	5.15	5.15	5.15
Soybean meal	6.40	6.30	6.20	6.10
Calcium monophosphate	0.30	0.30	0.30	0.30
NaCl	0.50	0.50	0.50	0.50
Premix ^2^	0.25	0.25	0.25	0.25
RPM	-	0.10	0.20	0.30
Zeolite	0.30	0.20	0.10	-
Total	100.00	100.00	100.00	100.00
Nutrient levels				
ME, MJ/Kg ^3^	8.73	8.74	8.74	8.74
Dry matter, %	89.49	89.15	89.21	89.30
Crude protein, %	12.51	12.49	12.50	12.47
Ca, %	0.86	0.86	0.86	0.86
P, %	0.32	0.32	0.32	0.32
NDF, %	51.53	51.55	51.55	51.53
ADF, %	25.54	25.59	29.59	25.57

^1^ NC, negative control diet (0 g/kg RPM); 1RPM, 2RPM, 3RPM: NC with 1, 2, or 3 g/kg of rumen-protected Met. ^2^ Per kg of premix provided the following: FeSO_4_·7H_2_O 170 g, CuSO_4_·5H_2_O 70 g, MnSO_4_·5H_2_O 290 g, ZnSO_4_·7H_2_O 240 g, CoCl_2_·6H_2_O 510 mg, KI 220 mg, Na_2_SeO_3_ 130 mg, VA 1 620 000 IU, VD3 324 000 IU, VE 540 IU, VK3 150 mg, VB1 60 mg, VB2 450 mg, VB12 0.9 mg. ^3^ ME was calculated value, while the other nutrient were measured values.

**Table 2:** Amino acid composition of diets fed to yearling Liaoning cashmere goats (DM basis, g/kg).

Item	Treatment ^1^			
	NC	1RPM	2RPM	3RPM
Methionine	3.42	4.13	4.79	6.03
Aspartic acid	14.83	15.01	15.01	15.02
Threonine	6.08	5.91	6.14	6.04
Serine	6.87	6.80	7.10	7.06
Glutamic acid	23.72	23.80	24.20	23.83
Glycine	7.22	7.02	7.14	7.09
Alanine	7.84	7.65	7.85	7.69
Cystine	17.42	17.46	17.32	17.48
Valine	13.20	13.00	12.97	12.72
Isoleucine	5.64	5.66	5.73	5.62
Leucine	10.68	10.31	10.71	10.55
Tyrosine	12.31	12.59	12.80	12.26
Phenylalanine	11.20	11.21	11.21	11.30
Lysine	8.84	8.69	8.79	8.91
Histidine	3.04	2.84	2.97	2.84
Arginine	9.21	9.13	9.01	9.28

^1^ NC, negative control diet (0 g/kg RPM); 1RPM, 2RPM, and 3RPM: NC diet with 1, 2, and 3 g/kg of rumen-protected Met, respectively.

**Table 3:** Effects of dietary rumen-protected methionine on growth performance of yearling Liaoning cashmere goats.

Items	Treatment ^1^				SEM ^2^	*p*-Values ^3^		
	NC	1RPM	2RPM	3RPM		Trt	Lin	Qua
BW, kg								
Initial	36.14	36.65	35.06	33.80	1.132	0.837	0.427	0.714
Final	43.60	45.40	46.20	42.40	1.160	0.678	0.811	0.255
ADG, g/d	70.64 ^b^	83.47 ^b^	106.13 ^a^	82.23 ^b^	3.470	<0.001	0.013	0.001
DMI, g/d	942	841	974	844	7.842	0.914	0.628	0.765
F/G ^4^	13.75 ^a^	10.10 ^b^	9.31 ^b^	10.37 ^b^	0.466	0.001	0.001	0.002

^1^ NC, negative control diet (0 g/kg RPM); 1RPM, 2RPM, and 3RPM: NC diet with 1, 2, and 3 g/kg of rumen-protected Met, respectively. ^2^ SEM = standard error of mean. ^3^ Trt = treatment; Lin = linear; Qua = quadratic. ^4^ F/G, the feed to gain ratio. ^a,b^ Mean values within a row with different superscripts differ significantly at *p* < 0.05.

**Table 4:** Effects of dietary rumen-protected methionine on nutrient intake and apparent digestibility by yearling Liaoning cashmere goats during faecal collection.

Items	Treatment ^1^				SEM ^2^	*p*-Values ^3^		
	NC	1RPM	2RPM	3RPM		Trt	Lin	Qua
Dry matter								
Intake, g/d	946	945	949	958	7.20	0.931	0.649	0.737
Digested, g/d	569	562	622	608	10.10	0.086	0.504	0.843
Digestibility, %	60.1 ^b^	59.5 ^b^	65.6 ^a^	63.4 ^ab^	0.89	0.036	0.607	0.619
Organic matter								
Intake, g/d	857 ^b^	871 ^b^	926 ^a^	868 ^b^	8.80	0.013	0.196	0.020
Digested, g/d	528 ^b^	587 ^a^	591 ^a^	565 ^ab^	9.10	0.046	0.130	0.016
Digestibility, %	61.7	67.4	63.8	65.1	0.79	0.064	0.313	0.133
Neutral detergent fibre								
Intake, g/d	488	487	489	494	3.70	0.931	0.649	0.737
Digested, g/d	265 ^b^	263 ^b^	300 ^a^	288 ^ab^	5.50	0.030	0.478	0.588
Digestibility, %	54.3 ^b^	53.9 ^b^	61.5 ^a^	58.2 ^ab^	1.06	0.027	0.608	0.442
Acid detergent fibre								
Intake, g/d	242	242	243	245	1.90	0.931	0.649	0.737
Digested, g/d	77.5	82.4	104.8	90.4	4.39	0.127	0.930	0.566
Digestibility, %	32.1	33.9	43.3	36.9	1.80	0.129	0.877	0.509

^1^ NC, negative control diet (0 g/kg RPM); 1RPM, 2RPM, and 3RPM: NC diet with 1, 2, and 3 g/kg of rumen-protected Met, respectively. ^2^ SEM = standard error of mean. ^3^ Trt = treatment; Lin = linear; Qua = quadratic. ^a,b^ Mean values within a row with different superscripts differ significantly at *p* < 0.05.

**Table 5:** Effect of dietary rumen-protected methionine on nitrogen utilisation by yearling Liaoning cashmere goats.

Items	Treatment ^1^				SEM ^2^	*p*-Values ^3^		
	NC	1RPM	2RPM	3RPM		Trt	Lin	Qua
Nitrogen								
Intake, g/d	18.90	18.90	18.90	19.20	0.150	0.931	0.649	0.737
Digestible N, g/d	12.62	13.67	13.91	14.31	0.246	0.081	0.015	0.477
Digestibility, %	66.60 ^b^	71.95 ^a^	73.54 ^a^	74.52 ^a^	1.051	0.024	0.005	0.235
Fecal excretion								
N, g/d	6.32 ^a^	5.32 ^b^	5.00 ^b^	4.87 ^b^	0.193	0.022	0.005	0.206
N/of N intake, %	33.40 ^a^	28.05 ^b^	26.46 ^b^	25.48 ^b^	1.051	0.024	0.005	0.236
Urinary excretion								
N, g/d	5.85	5.61	5.52	5.41	0.319	0.972	0.646	0.928
N/of N intake, %	30.67	29.33	29.17	28.17	1.563	0.962	0.612	0.961
Retention								
N, g/d	6.77	8.05	8.39	8.89	0.346	0.157	0.032	0.561
N/of N intake, %	35.84	42.54	44.47	46.33	1.846	0.206	0.048	0.503
N/of digestible N, %	53.67	59.09	60.53	62.04	2.234	0.601	0.209	0.674
Plasma								
Urea N, mmol/L	3.99 ^a^	2.73 ^b^	1.96 ^c^	2.91 ^b^	0.188	<0.001	0.001	<0.001

^1^ NC, negative control diet (0 g/kg RPM); 1RPM, 2RPM, and 3RPM: NC diet with 1, 2, and 3 g/kg of rumen-protected Met, respectively. ^2^ SEM = standard error of mean. ^3^ Trt = treatment; Lin = linear; Qua = quadratic. ^a–c^ Mean values within a row with different superscripts differ significantly at *p* < 0.05.

**Table 6:** Effect of dietary rumen-protected methionine on plasma free amino acids of yearling Liaoning cashmere goats (ng/mL).

Items	Treatment ^1^				SEM ^2^	*p*-Values ^3^		
	NC	1RPM	2RPM	3RPM		Trt	Lin	Qua
Methionine	3.52 ^c^	6.45 ^b^	7.20 ^ab^	7.40 ^a^	0.351	<0.001	<0.001	<0.001
Phenylalanine	10.50	9.49	8.90	9.41	0.227	0.077	0.049	0.080
Lysine	27.98	24.49	21.70	20.22	1.281	0.144	0.025	0.678
Threonine	44.41	38.62	36.36	34.30	3.581	0.797	0.342	0.806
Arginine	31.67	30.18	28.46	28.31	1.068	0.674	0.245	0.766
Histidine	11.82	8.02	9.54	7.34	1.165	0.565	0.276	0.740
Leucine	20.75 ^a^	15.09 ^b^	15.01 ^b^	13.97 ^b^	0.821	0.006	0.002	0.091
Isoleucine	14.42 ^a^	11.22 ^b^	8.36 ^c^	7.90 ^c^	0.688	<0.001	<0.001	0.146
Valine	29.25	26.06	25.17	24.92	0.791	0.190	0.053	0.340
Glutamic acid	33.28	28.93	29.66	28.46	1.186	0.492	0.215	0.518
Aspartic acid	5.48	5.10	4.90	5.09	0.266	0.906	0.595	0.620
Proline	27.90	26.67	25.62	25.66	0.620	0.403	0.099	0.792
Glycine	91.72	81.37	81.04	79.81	1.186	0.485	0.195	0.457
Serine	15.78	12.55	12.03	11.94	0.878	0.376	0.139	0.380
Alanine	20.86	22.35	17.83	19.15	0.692	0.098	0.106	0.947
Cystine	16.12	14.06	13.83	13.41	0.489	0.209	0.060	0.392
Tyrosine	18.69	17.51	16.73	18.15	0.489	0.551	0.592	0.206
EAA ^4^	194.32	169.62	160.68	153.77	6.442	0.122	0.024	0.467
NEAA ^5^	229.83	208.54	201.64	201.66	4.486	0.068	0.018	0.215
BCAA ^6^	64.42 ^a^	52.37 ^b^	48.54 ^b^	46.80 ^b^	2.166	0.008	0.002	0.154
TAA ^7^	424.15 ^a^	378.17 ^ab^	362.32 ^b^	355.44 ^b^	9.730	0.040	0.008	0.272

^1^ NC, negative control diet (0 g/kg RPM); 1RPM, 2RPM, and 3RPM: NC diet with 1, 2, and 3 g/kg of rumen-protected Met, respectively. ^2^ SEM = standard error of mean. ^3^ Trt = treatment; Lin = linear; Qua = quadratic. ^4^ EAA = Essential AA = Met + Phe + Lys + Thr +Arg + His + Leu + Ile + Val. ^5^ NEAA = Nonessential AA = Glu + Asp + Pro + Gly + Ser + Ala + Cys + Tyr. ^6^ BCAA = Branched-chain AA = Ile + Leu + Val. ^7^ TAA = Total amino acids. ^a–c^ Mean values within a row with different superscripts differ significantly at *p* < 0.05.

## Data Availability

The datasets collected and analysed during the current study are available on fair request from the corresponding author.
